# Complete Genome Sequence of *Bacillus thuringiensis* subsp. *kurstaki* Strain HD73

**DOI:** 10.1128/genomeA.00080-13

**Published:** 2013-03-14

**Authors:** Guiming Liu, Lai Song, Changlong Shu, Pinshu Wang, Chao Deng, Qi Peng, Didier Lereclus, Xumin Wang, Dafang Huang, Jie Zhang, Fuping Song

**Affiliations:** a State Key Laboratory for Biology of Plant Diseases and Insect Pests, Institute of Plant Protection, Chinese Academy of Agricultural Sciences, Beijing, China; b CAS Key Laboratory of Genome Sciences and Information, Beijing Institute of Genomics, Chinese Academy of Sciences, Beijing, China; c INRA, UMR1319 Micalis, Génétique Microbienne et Environnement, La Minière, Guyancourt, France

## Abstract

*Bacillus thuringiensis* is a Gram-positive bacterium that produces intracellular protein crystals toxic to a wide variety of insect larvae. We report the complete genome sequence of *Bacillus thuringiensis* subsp. *kurstaki* strain HD73 from the Centre OILB (Institut Pasteur, France), which belongs to serotype 3ab and is toxic to lepidopteran larvae.

## GENOME ANNOUNCEMENT

*Bacillus thuringiensis* is a Gram-positive bacterium that produces intracellular protein crystals (cry) toxic to a wide variety of insect larvae and is the most commonly used biological pesticide (1). Many *B. thuringiensis* strains contain multiple *cry* genes, harbored in plasmids (2). *Bacillus thuringiensis* subspecies *kurstaki* strain HD73 (*B. thuringiensis* HD73), toxic to lepidopteran larvae (serotype 3ab) (3), contains large self-transmissible plasmids, including pHT73 and pAW63 (4). This strain was obtained from the Centre OILB (Institut Pasteur, France). This strain was provided as the *B. thuringiensis* subspecies *kurstaki* type strain and was also designated the *B. thuringiensis* subspecies *kurstaki* KT0 strain (5). Only one endotoxin gene, the *cry1Ac* gene, was found to be harbored in pHT73 (6–8), and this gene was considered to be a typical example of a sporulation-dependent crystal gene because the *cry1A*-like promoter is controlled by sigma E and sigma K during sporulation (9, 10). However, weak transcription of this promoter was detected in the nonsporulating cell (11).

Here, we determined the whole genome sequence to obtain the genomic information. Genomic DNA was isolated from *B. thuringiensis* HD73. Genome sequencing was performed with 454 GS-FLX titanium (Roche Applied Science) and Illumina GAII (Illumina, United States) platforms. A total of 167,017 high-quality Roche 454 reads with an average read length of 412 bp were produced, providing about 12-fold coverage of the genome, while the Illumina reads provide 370-fold coverage with 21.7 million reads of 100 bp (with insert size 8,000 bp). After preprocessing, Roche 454 reads were assembled into contigs with Newbler, version 2.6, and then scaffolded with Illumina mate-pair reads using SSPACE (12). A 40-kb fosmid library was constructed and subjected to bidirectional end sequencing of 2,261 clones. The gaps were closed by PCR amplification and primer walking. The open reading frames (ORFs) were identified by using Glimmer version 3.02 (13). The tRNAs and rRNAs were predicted using tRNAscan-SE (14) and RNAmmer (15), respectively. The functions of encoding genes were annotated by using NCBI nr, COG (clusters of orthologous groups) (16), KEGG (17), and InterProScan (18).

The genome of *B. thuringiensis* HD-73 contains 8 replicons, including a circular chromosome and 7 plasmids. The chromosome was 5.6 Mb in length with a GC content of 31.4%, containing 5,892 protein-encoding genes, 104 tRNA, and 36 rRNA-encoding operons. Approximately 32.1% of all coding sequences (a total of 1,894) were assigned to COGs, and 1,282 CDSs can be annotated into the 1109 KEGG orthology system by using KAAS (19). The 7-plasmid length ranges from 8 kb to 77 kb, with GC content from 29.73% to 34.66%, carrying a total of 235 ORFs. Besides the self-transmissible plasmid pHT73, our data revealed another large plasmid, pHT77, in addition to those revealed in a previous study (4), because the size similarity makes it difficult to distinguish these plasmids by gel electrophoresis (pHT77 of 76,490 bp and pHT73 of 77,351 bp). The genome sequence provides insights into plasmid conjugation, spore formation, crystal formation, virulence factor interaction with insect host, and evolution of *B. thuringiensis*.

### Nucleotide sequence accession numbers.

The annotated chromosome and plasmids have been deposited in GenBank under the accession numbers: CP004069 (chromosome), CP004070 (pHT73), CP004071 (pHT77), CP004073 (pHT11), CP004074 (pHT8_1), CP004075 (pHT8_2), and CP004076 (pHT7).
